# 1412. Racial Differences in Candidemic Patients at a Southern California Teaching Hospital

**DOI:** 10.1093/ofid/ofac492.1241

**Published:** 2022-12-15

**Authors:** Victoria C Grant, Anna Y Zhou, Jacinda C Abdul-Mutakabbir, Karen K Tan

**Affiliations:** Loma Linda University, Rancho Cucamonga, California; Loma Linda University Medical Center, RANCHO CUCAMONGA, California; Loma Linda University, Rancho Cucamonga, California; Loma Linda University Medical Center, RANCHO CUCAMONGA, California

## Abstract

**Background:**

Racial disparities in diagnosis and management of infectious diseases in the US healthcare system have been reported. Candidemia is a leading healthcare associated bloodstream infection and is associated with significant mortality. Further, global rates of azole-resistant *C. parapsilosis* have dramatically increased. Nonetheless, literature describing racial and ethnic differences among candidemic patients is limited. The objective of this study was to describe infection characteristics and outcomes among non-Hispanic White (n-REM) and racially and ethnically minoritized (REM) patients with candidemia.

**Methods:**

Adult patients hospitalized with ≥ 1 positive blood culture growing *Candida* between 1/2020 and 12/2021 were included. Based on documented race and ethnicity, patients were dichotomized into the n-REM or REM group. Pertinent data was collected and then compared between groups using univariate analysis. Significance was defined as *P* ≤0.05.

**Results:**

86 unique episodes of candidemia were included. REM patients were significantly younger (mean age 54 vs 62 years; *P* = 0.017) and had more risk factors for candidemia (median 4 vs 3). REM patients were also more likely to present with sepsis/septic shock (87% vs 81%) and require an ICU stay (57% vs 53%). Time to detection of candidemia was shorter in the REM group (median 38.5 vs 43 hours); however, time to initiation of active antifungal therapy was longer (median 48 vs 44 hours). Additionally, hospital LOS was longer in the REM group (median 25.5 vs 19.5 days). Central line infection was the most common source of candidemia. Despite similar documented sources of infection, microbiological differences were noted. *Candida glabrata* was more often isolated in n-REM patients (50% vs 27%; *P* = 0.04), whereas *C. parapsilosis* was more often isolated in REM patients (17% vs 3%; *P* = 0.048).

**Conclusion:**

Despite being younger, REM patients were at an increased risk for candidemia. Furthermore, *C. parapsilosis*, a globally growing species of concern due to azole resistance, was more frequently isolated among REM patients. Rapid detection and speciation of candidemia may be particularly important among REM patients to ensure early initiation of optimal therapy. Further research exploring racial differences among candidemic patients is needed.

**Disclosures:**

**All Authors**: No reported disclosures.

